# Surgery

**Published:** 1900-06

**Authors:** John Parmenter, Nelson W. Wilson

**Affiliations:** Buffalo, N. Y., Professor of anatomy and adjunct professor of clinical surgery, Buffalo University Medical College; Buffalo, N.Y., Acting Assistant Surgeon United States Army


### Progress in Medical Science.

### SURGERY.

CONDUCTED BY JOHN PARMENTER, M. D., Buffalo, N. Y.,
Professor of anatomy and adjunct professor of clinical surgery, Buffalo University Medical College.
AND
NELSON W. WILSON, M. D., Buffalo, N. Y.
Acting Assistant Surgeon United States Army.

### THE GOOD OF THE POULTICE.

THERE has been so much general and popular misuse made of poultices where they were and were not indicated, irrespective of time, place or condition, that the old friend and standby has been brought into such general disrepute that if it is not wholly discarded its benefits, at least, have been lost sight of in many, many cases. S. E. Earp, writing on this subject in the New York *Med. Jour.*, says that the active principles of the poultice are moisture and heat and hence it matters little what they consist of, except so far as their capability of retaining heat is concerned. They must be generous in size, applied hot, and frequently changed. In general it is better to use antiseptic fomentations upon open wounds, but the charcoal poultice is of considerable value as a deodorant in some cases of foul ulcer. The virtues of a poultice are seen in its power (1) to relieve congestion, (2) to promote absorption, favor resolution and hasten suppuration, (3) to diminish tension, (4) to soften incrustations, (5) to stimulate healthy granulations and (6) to perform the office of a deodorant.

### LOCAL ANESTHESIA WITH EUCAINE B.

IN THE *Lancet*, A. E. Barker reports fifty-three cases, including radical cure of hernia, resection of intestine, gastro-enterostomy, goitre, wiring of patella, and the like, in which local anesthesia by means of 1 to 1000 eucaine B. in normal salt solution was used. For some patients as much as six ounces of this solution were used with no ill-effect. There is no interference with primary union. Children and timid adults are not adapted to its use, for they are frightened by the surgical surroundings. Acutely inflamed areas cannot be rendered entirely anesthetic. Dragging the mesentery causes a sense of pressure or griping.

### LEGRAND'S SOLUTION ANESTHÉSIQUE-HÉMOSTATIQUE.

THIS solution can be sterilised and kept for a long time without in any way deteriorating. The method of using is the same as for Schleich's cocaine infiltration anesthesia. The special claim made for this solution is that there is an entire absence of secondary vaso-motor dilatation following the primary contraction produced by the cocaine. The hemostatic agent is the gelatin. The formula is as follows:

| | | |
|---|---|---|
| ℞ | Gelatin. pur. . . . . . . . . . . . . . . . . . . . | 2 0 |
| | Sodii chloridi . . . . . . . . . . . . . . . | 0.7 |
| | Acidi carbol. cryst. . . . . . . . . . . . . . . . . | 0.1 |
| | Eucain. hydrochlor. B. . . . . . . . . . . . . . . . | 0 7 |
| | Cocain. hydrochlor. . . . . . . . . . . . . . . | 0.3 |
| | Aquæ distill . . . . . . . . . . . . . . . . . . . . ad | 100 0 |
| M. | | |

### THE USE OF SALT SOLUTION.

J. WESLEY BOVEE, writing of the use of decinormal salt solution says: "Ordinarily not more than one ounce per minute should be injected into the tissues or vein. Pulmonary edema, dyspnea, headache, vertigo, mental excitement, delirium, hallucinations, severe pain in the left side, with engorgement of the liver and spleen, occur from overdistention of the bloodvessels with salt solution."

### GUAIACOL AS AN ANESTHETIC.

LUCAS CHAMPIONNIÈRE says that guaiacol in solution in oil is used as a local anesthetic, especially for the turbinals and for the removal of polypi in the nose and ears. Anesthesia is obtained in ten minutes. Eucaine, which is less poisonous than cocaine, is used in a solution of 5 to 8 per cent.

### TO THREAD A NEEDLE RELIGIOUSLY.

ONE of the most prolific causes of what might properly be called surgical profanity is the difficulties surrounding the threading of needles. Dr. Joseph M. Jackson has entered the missionary field and has pointed out the straight and narrow path to those whose patience has been tried and whose profanity has been lurid while engaged in threading a needle. Here's the anti-swear method: "To thread a needle hold it with the ring and little fingers of the left hand, instead of with the thumb and forefinger as is the usual way. This method leaves the thumb and forefinger free to grasp the smallest bit of silk or other suture material as it passes through the eye and pull it to a safe distance on the other side. This does away with the slipping back, so common in the old way of changing hands."

### TO PRESERVE NEEDLES.

DAWBARN says keep them in a saturated solution of washing soda. Carbolic oils and watery solutions dull all cutting instruments, and in alcohol they will soon rust. Albolene has an unpleasant oiliness but is otherwise good. Calcium chloride in absolute alcohol is efficacious but expensive.

### THE APPENDIX AND THE KNIFE.

DR. CARL BECK looks on all appendices as evils, and in summing up the whole question of operation says: "However light the clinical expression of appendicitis may be, and how much it may appear to be in favor of a speedy temporary recovery, the operation is always justifiable. As the strength of the infection can never be known with certainty from the beginning, it appears to be wiser to take each appendicitis seriously. Among two evils the smaller should be chosen, and operation is the smaller evil."

---

TO EXTRACT FOREIGN BODIES FROM THE NOSE.

FELIZET makes a very comon-sense suggestion regarding the removal of foreign bodies from children's noses as follows : "Inject through the sound nostril a current of warm salt water at a moderate pressure, which, by returning by the posterior nares on the occluded side, forces the foreign body out, or at least allows of its being seized with forceps."

